# Evaluating diagnostic strategies for early detection of cancer: the CanTest framework

**DOI:** 10.1186/s12885-019-5746-6

**Published:** 2019-06-14

**Authors:** Fiona M. Walter, Matthew J. Thompson, Ian Wellwood, Gary A. Abel, William Hamilton, Margaret Johnson, Georgios Lyratzopoulos, Michael P. Messenger, Richard D. Neal, Greg Rubin, Hardeep Singh, Anne Spencer, Stephen Sutton, Peter Vedsted, Jon D. Emery

**Affiliations:** 1 The Primary Care Unit, Department of Public Health & Primary Care, University of Cambridge, Cambridge, CB1 8RN UK; 2 Department of Family Medicine, University of Washington, Seattle, USA; 3 University of Exeter, St Luke’s Campus, Exeter, EX1 2LU UK; 4 Department of Behavioural Science and Health, Epidemiology of Cancer Healthcare and Outcomes (ECHO) Research Group, University College London, London, UK; 5 National Institute of Health Research (NIHR) Leeds In Vitro Diagnostic Cooperative (IVDC), Leeds Centre for Personalised Medicine and Health, University of Leeds, Leeds, UK; 6 Academic Unit of Primary Care, Leeds Institute of Health Sciences, University of Leeds, Leeds, UK; 7 Institute of Health and Society, University of Newcastle, Sir James Spence Institute, Royal Victoria Infirmary, Newcastle, NE1 4LP UK; 8 Center for Innovations in Quality, Effectiveness and Safety, Michael E. DeBakey Veterans Affairs Medical Center and Baylor College of Medicine, Houston, TX USA; 9 Health Economics Group, University of Exeter, St Luke’s Campus, Exeter, EX1 2LU Devon UK; 10 Research Centre for Cancer Diagnosis – CaP, The Research Unit for General Practice and Research Clinic for Innovative Health Care Delivery, Department of Clinical Medicine, Aarhus University, Bartholins Alle 2, 8000 Aarhus, Denmark; 11 Centre for Cancer Research and Department of General Practice, University of Melbourne, 10th floor, Victorian Comprehensive Cancer Centre, 305 Grattan St, Melbourne, VIC 3010 Australia

**Keywords:** Cancer, Diagnostic strategies, Early detection, Diagnosis, Conceptual framework, Primary care

## Abstract

**Background:**

Novel diagnostic triage and testing strategies to support early detection of cancer could improve clinical outcomes. Most apparently promising diagnostic tests ultimately fail because of inadequate performance in real-world, low prevalence populations such as primary care or general community populations. They should therefore be systematically evaluated before implementation to determine whether they lead to earlier detection, are cost-effective, and improve patient safety and quality of care, while minimising over-investigation and over-diagnosis.

**Methods:**

We performed a systematic scoping review of frameworks for the evaluation of tests and diagnostic approaches.

**Results:**

We identified 16 frameworks: none addressed the entire continuum from test development to impact on diagnosis and patient outcomes in the intended population, nor the way in which tests may be used for triage purposes as part of a wider diagnostic strategy. Informed by these findings, we developed a new framework, the ‘CanTest Framework’, which proposes five iterative research phases forming a clear translational pathway from new test development to health system implementation and evaluation.

**Conclusion:**

This framework is suitable for testing in low prevalence populations, where tests are often applied for triage testing and incorporated into a wider diagnostic strategy. It has relevance for a wide range of stakeholders including patients, policymakers, purchasers, healthcare providers and industry.

**Electronic supplementary material:**

The online version of this article (10.1186/s12885-019-5746-6) contains supplementary material, which is available to authorized users.

## Background

Diagnosing cancer early is a public and policy priority, with primary care the preferred setting for this to occur [1]. This has led to a desire for better tests for early detection of cancer, ideally ones useable in primary care. However, sustained and substantial investment in the development of novel biomarkers and other tests has mainly benefited prognostication and surveillance of patients already diagnosed with the disease [2]. In contrast, there have been few benefits in improving the precision and timeliness of diagnosis of cancer in cancer patients who generally present to primary care with symptoms [3]. A wider range of better tests could be transformational [4]. This ‘grand challenge’ of improving early cancer diagnosis has been recognised by a wide range of stakeholders including policymakers, purchasers, health care providers and consumers, and industry.

While advances in technology are producing a growing array of new diagnostics involving biomarkers, sensors, imaging devices and artificial intelligence algorithms [5], the vast majority of apparently promising cancer diagnostic tests in early development fail because they do not perform adequately in the low prevalence populations in which they will eventually be applied [6], the so-called ‘spectrum effect’. Thus, a test developed in a population with a higher prevalence of disease (or at higher risk) will typically have a lower sensitivity and higher specificity when applied in a population with lower disease prevalence (or at lower risk) [6]. This leads to high rates of false positive tests, and increasing referrals to specialist care, such as symptomatic women with raised CA125. Alternatively, a test may be marketed too soon and be inappropriately applied – the implementation of Prostate Specific Antigen (PSA) testing at population level, even before results of screening trials were available, is a well-known example [7]. Thus, the challenges of evaluating tests for cancer and other low-prevalence conditions include potential over-investigation and over-diagnosis, deciding on the reference standards to be used in assessing test accuracy, and outcomes relevant to patients.

Rigorous evaluation of new tests has been undertaken across a range of medical disciplines such as biochemistry, pathology, radiology and genomics, informed by frameworks developed by academic or policy groups at national or international levels. These frameworks apply at various stages in the diagnostic pathway from early development to implementation. They aim to guide a variety of stakeholders, including test developers, clinicians, researchers and policy makers, on what evidence is needed at each stage of development of a test ‘from bench to community’.

The last review of diagnostic test frameworks, published in 2009, identified several common phases of test evaluation: technical efficacy, clinical accuracy, comparative accuracy, diagnostic and therapeutic impact, patient outcomes, and societal aspects [8]. Most frameworks described only parts of the diagnostic evaluation process but many failed to consider issues specific to populations with a low prevalence for the condition of interest.

We aimed to address this. Specifically, we were interested in frameworks with explicit recognition of:

(i) the prevalence of cancer in the studied population and potential impact of spectrum bias [6];
(ii) the application of a test as a triage test rather than a diagnostic test to raise post-test probability of cancer to inform subsequent decision making and definitive testing; and
(iii) the incorporation of a test into a broader diagnostic strategy i.e. involving either more than one test undertaken concurrently or sequentially, or integrated into a more complex approach using the test result combined with other data (e.g. demographics, symptoms, simple measurements such as BMI, and other tests) within the evaluation framework.

We call these ‘essential criteria’ from now on.

## Methods

### Systematic scoping review

**Fig. 1:**
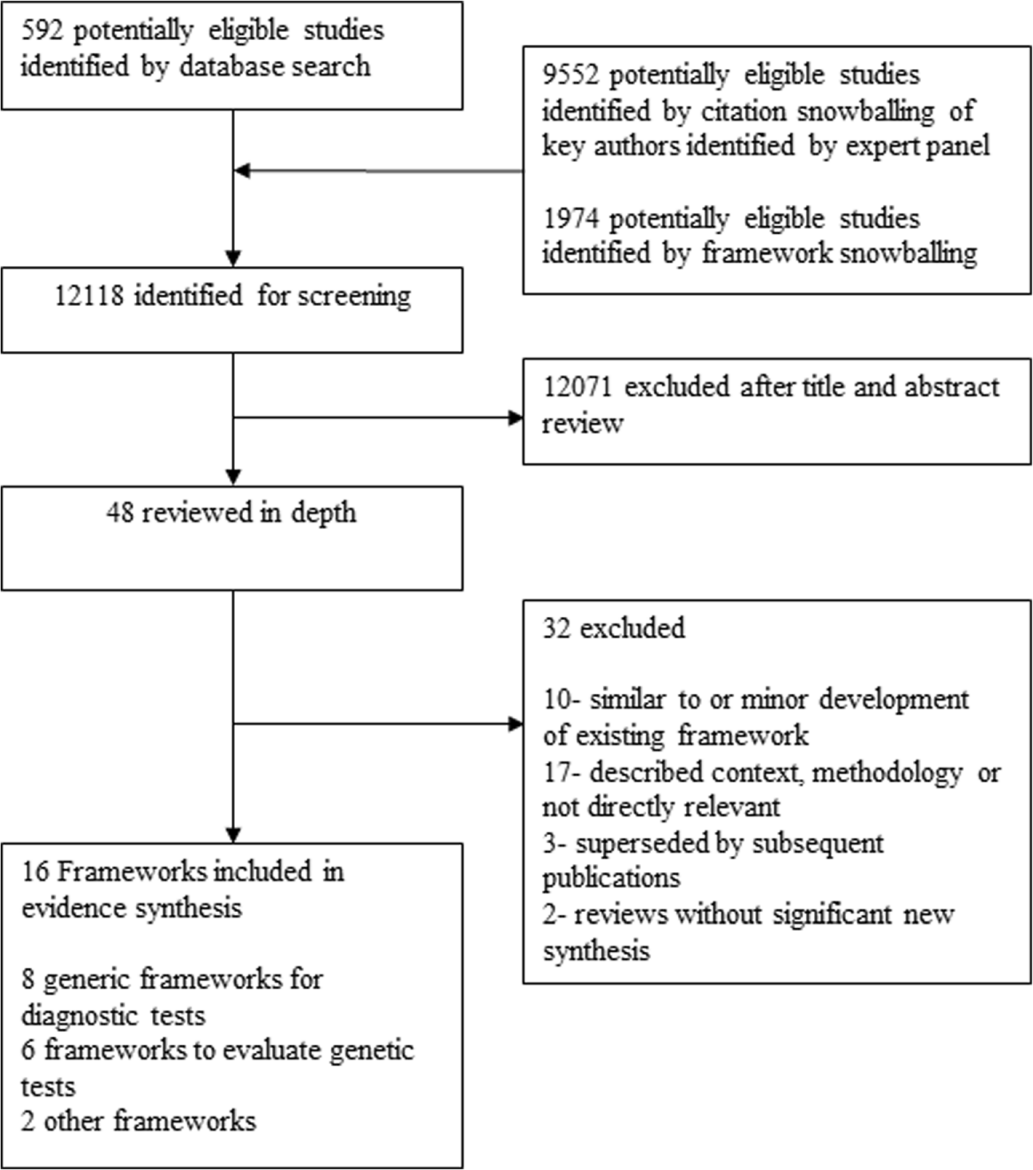
Flow Diagram for systematic scoping review

We undertook a systematic scoping review to examine prior theoretical frameworks using the Arksey and O’Malley six-stage methodological framework, enhanced by more recent recommendations (see Fig. 1) [9, 10]. Our search strategy was initially based on Lijmer et al’s search terms [8]. We conducted searches for literature published from January 2009 to August 2017 in Medline, Embase and Web of Science (*n* = 592), and asked the consensus group to identify relevant frameworks and authors active in the field of diagnostic test evaluation. We combined the results to form a body of literature, further investigated using retrospective and prospective snowball methods, to search reference lists of framework papers published since the Lijmer et al. review, and citations of framework papers and authors’ papers published in the past 10 years (*n* = 9552). We then selected for inclusion, models or frameworks for the evaluation of medical tests, at any stage in the diagnostic pathway, published in a peer-reviewed journal, and in the English language (*n* = 47 for full-text review).

We included 16 frameworks (see Table 1 and Additional file 1: Table S1). The included frameworks focused on generic frameworks for diagnostic tests (*n* = 8) [11–18]; frameworks specific to the evaluation of genetic tests (*n* = 6) [5, 19–23]; a framework addressing issues of diagnostic safety and the use of the test within the context of the patient-doctor consultation (*n* = 1) [24]; and on value proposition of testing (*n* = 1) [25]. They were mostly developed by authors in North America, and frequently referenced Fryback & Thornbury’s original framework paper, the Hierarchical Model of Efficacy, published in 1991 [11]; many of the subsequent frameworks describe similar phases of research to demonstrate these levels of evidence. We reviewed these preliminary findings at an international consensus meeting, held in London, October 2017, and were unable to identify a single framework that recognized the three essential criteria. Specifically, the limitations of existing frameworks were: usually focusing on a single test rather than a series of tests or a comparator test; not recognising that some tests can be used to raise post-test probability of cancer to inform selection for subsequent definitive diagnostic testing i.e. use as a triage test, and; mostly not accounting for incorporation of the test into a diagnostic strategy.

**Table 1:** Summary of included frameworks (see Additional file 1: Table S1 for further information)

	Authors Year & setting	Framework	Field	Cancer-specific	Low prevalence population	Triage test	Diagnostic strategy
GENERIC TEST FRAMEWORKS *n* = 8							
1	Fryback & Thornbury [12], 1991, USA	*Hierarchical Model of Efficacy*	Imaging	No	No	Yes	No
2	Harris et al. [14] 2001, USA	*U.S. Preventive Services Task Force*	Generic	No	Yes	No	N/S
3	Pepe et al.[15] 2001, USA	Phases of biomarker development for early detection of cancer	Laboratory medicine	Yes	No	No	No
4	Gazelle et al. [15] 2011, USA	*Framework for assessing the vaue of diagnostic imaging*	Imaging	No	No	No	N/S
5	Febbo et al. [13] 2011, USA	*Evaluating the clinical utility of tumor markers in oncology.* NCCN Task Force Report	Oncology	Yes	No	No	No
6	Ferrante Di Ruffano et al. [16] 2012, UK and international partners	*Framework for designing and evaluating trials.*	Generic	No	No	Yes	Yes
7	Horvath et al. [17] 2014, Australia	*From biomarkers to medical tests: The changing landscape of test evaluation.*	Laboratory medicine	No	No	Yes	Yes
8	Thompson et al. [18] 2016, USA	*Framework to incorporate multiple test attributes in evaluating diagnostic tests including Point-of-Care tests.*	Generic	No	Yes	N/S	N/S
TESTS SPECIFIC TO EVAULATING GENETIC TESTS *n* = 6							
9	Phillips et al. [4] 2006 USA	*Diagnostics and biomarker development: priming the pipeline.*	Pharmacogenetics	No	No	No	No
10	Teutsch et al. [19] 2009, USA	*Evaluation of Genomic Applications in Practice and Prevention- the EGAPP Framework.*	Genetics	No	Yes	No	No
11	Rosenkötter et al. [20] 2011, European	*The Contribution of Health Technology Assessment, Health Needs Assessment, and Health Impact Assessment to the Assessment and Translation of Technologies in the Field of Public Health Genomics.*	Genetics	No	N/S	No	No
12	Rousseau et al. [21] 2010, Canada	*Development and description of GETT: a Genetic testing Evidence Tracking Tool.*	Genetics	No	N/S	N/S	No
13	Sun et al. [22] 2013, USA	*Evaluation frameworks and assessment of analytic validity.*	Genetics	No	N/S	No	N/S
14	Lin et al. [23] 2012, USA, UK	*Evaluating genomic tests from bench to bedside: a practical framework.*	Genetics	No	Yes	No	N/S
OTHER FRAMEWORKS *n* = 2							
15	Singh & Sittig [24], 2015, USA	*Safer Dx Framework.*	Generic	No	N/S	Yes	Yes
16	Price & St John [25], 2014, UK & international partners	*Anatomy of value proposition for laboratory medicine.*	Laboratory medicine	No	N/S	Yes	Yes

### Synthesis and development of the CanTest framework

Although no framework fully met our requirements, the consensus group agreed that the Lin et al model mapped most closely to our aims [23]. However, it omitted key aspects of incorporation of a test into a testing strategy, and the usage of a test for triage. Furthermore, most of the frameworks were too simplistic, and ignored non-linearity in development, that is, the need for iteration back and forth between phases of research. Horvath et al’s Test Evaluation Framework, [17] and Thompson et al’s model incorporating multiple test attributes [18], were among the few which recognised the iterative or cyclical nature of test evaluation and interplay between different phases of evaluation. The SaferDx framework was the only one to deal in detail with the interplay of the test’s performance and the provider interpretation within the wider context of the patient’s diagnostic process, as well as interactions between various components of the diagnostic work system distributed in space and time [24]. SaferDx also made explicit the role of the test in triage of patients for possible additional testing and speciality referral. Finally, SaferDx recognised the need to coordinate the diagnostic process (often involving performance and interpretation of different tests at different times and locations) and to ensure fail-safe patient follow-up.

As no existing framework was satisfactory, the consensus group developed the CanTest Framework, informed by these key papers, and refined by further iterative discussion and consensus within the multidisciplinary group. We aimed to develop a new comprehensive, methodological framework that addressed the continuum from development of the test to impact on diagnosis and patient outcomes in routine practice, for use by test developers, including industry, research funders and academics. We specifically aimed to incorporate: a shift in focus away from a single test towards evaluation of its integration into a diagnostic strategy; greater clarity around the changes in test performance from highly selected populations towards the final intended, lower prevalence population; and the iterative nature of test evaluation and development.

## Results

**Fig. 2:**
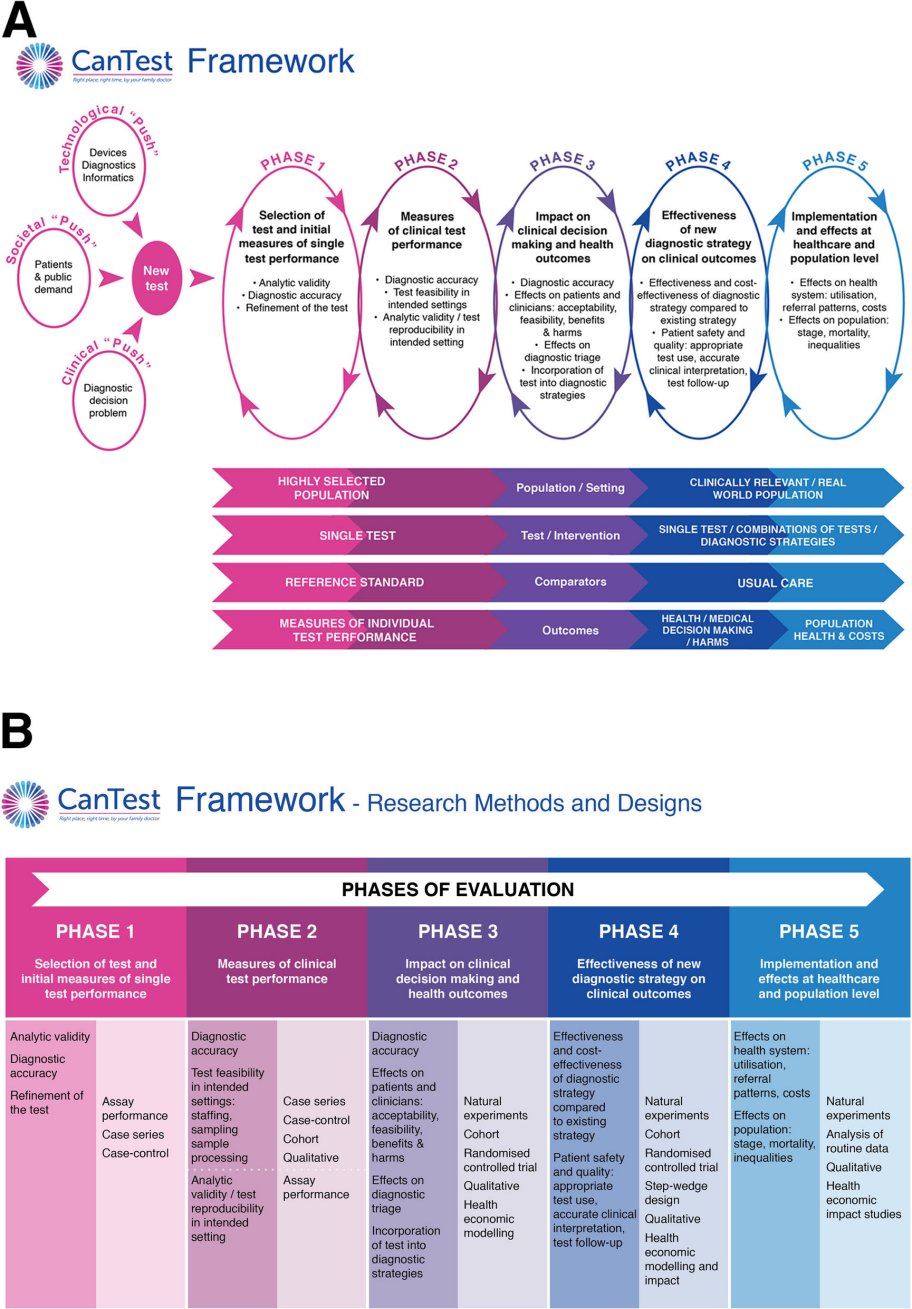
**a** The CanTest Framework. **b** The CanTest Framework - Design and Methods

Figure 2a depicts the core elements of the CanTest Framework. The consensus group chose to focus on the translational pathway of tests for which there was already preliminary evidence of analytic validity [26]. Importantly, the CanTest Framework is cyclical, reflecting the iterative nature of translational research and how failure to establish important developmental steps returns the test evaluation to a previous phase, and potential redesign of the test, as well as forward to the next phase.

The CanTest Framework consists of two elements. The upper part of the figure depicts the five phases that a new test ideally should follow (whether ‘pushed’ by patient and public demand or technological development, or ‘pulled’ by clinical need) before implementation into routine practice. The lower part of the figure outlines the changes that occur during these phases in the population/setting, the test and its incorporation into a diagnostic strategy, the test comparators, and the outcomes measured (the so-called ‘PICO’ elements summarising ‘population, intervention, comparator and outcomes’). Across the framework key issues of bias and generalisability are relevant to the overall clinical validity of a new test. This includes, for example, potential overfitting of data in Phase 1, bias due to known and unknown differences between comparator groups, and bias due to retrospective designs [27]. Spectrum bias, an issue of generalisability, is also critical across the framework, hence the explicit consideration of study population, disease prevalence and the final intended population in which the test will be used.

**Phase 1- Selection of test and initial measures of single test performance,** typically occurring at the start of test evaluation. It includes measures of analytic validity, diagnostic accuracy, and often some technical refinement of the test. At this stage, the test is evaluated in a highly selected population in ‘proof of principle’ studies, against a reference or gold standard, and focusses exclusively on comparative performance of that individual test to a reference standard.

**Phase 2- Measures of clinical test performance,** provides information on diagnostic accuracy, and the feasibility of performing the test in intended populations and settings (such as staffing needs, sampling, processing). In addition, it provides further evidence about analytic validity and test reproducibility in the intended settings. At this phase, tests are still evaluated in relatively selected populations and therefore with a higher prevalence of cancer than in the final intended population. The test is again likely to be evaluated alongside, and in comparison to, a reference standard in potentially several comparative accuracy studies, of increasing generalisability.

**Phase 3- Impact on clinical decision-making and health outcomes,** provides information on diagnostic accuracy in intended populations, and begins to examine measures of clinical utility focusing on the impact of the test on clinicians and patients. This includes acceptability and feasibility of the test from both perspectives, its impact on clinical decision-making, diagnostic triage and incorporation into diagnostic strategies. By this phase, the population in which the test is being evaluated is becoming more similar to the final intended population, and the test may be evaluated as part of a combination of tests. The reference standard to which the test is being compared may now have changed to usual care rather than an ideal or perfect gold standard in comparative accuracy studies. Outcomes are no longer restricted to test performance, but rather also include those related to clinical decision-making and patient experience, including quantifying benefits and harms.

**Phase 4- Effectiveness of new diagnostic strategy on clinical outcomes,** evaluates the effectiveness and cost-effectiveness of the new diagnostic strategy compared to existing strategy(s). The key changes are the test is now being evaluated in the population in which it is intended to be used, and it is more likely to be evaluated as part of a combination of tests or test strategies. The comparison is now firmly with usual care testing processes, and the outcomes measured are those of clinical and cost-effectiveness.

Ideally, tests should only be implemented following Phase 4 evaluations, but pre-implementation assessment can occur from Phase 3 and, even after adoption into routine practice, a further phase of post-implementation evaluation is important.

**Phase 5- Implementation and effects at health care system and population level.** Post-implementation surveillance should determine effects on the health care system, including use of the test beyond the intended population, referral patterns and costs, as well as effects at the population level such as stage at diagnosis, survival and inequalities in the use of diagnostic care and relevant clinical outcomes. At this stage, the test or diagnostic strategy is often evaluated using routine observational data or qualitative studies.

Figure 2b has been included as it complements the CanTest Framework with additional guidance on research design and methods most appropriate for each phase of evaluation of a test or diagnostic strategy. Small-scale efficacy trials have a role from Phase 3 moving towards larger pragmatic randomised controlled trials in phase 4. These would be complemented by other research methods, such as qualitative approaches, health economic modelling and impact studies, and natural experiments arising from premature implementation.

## Discussion

Diagnostic test studies generally focus on accuracy, often in a population already diagnosed with cancer, yet a clear translational pathway from new test development to health system implementation requires a wider assessment of their value and impact on patients and the healthcare system, including cost-effectiveness, crucially in the population for which the test is intended [16]. Without a more efficient (or clearly laid out) pathway, there is a risk that the pathway to implementation is not only slow but also unattractive to investors and research funders. Indeed, a recent review of new diagnostic tests in primary care settings showed that the median time to complete the ‘invention to implementation’ cycle was 9 years (IQR 6–13 years) [28]. A series of papers published in 2017 on decision-making about healthcare-related tests and diagnostic test strategies started with the acknowledgement that ‘surprisingly little progress has been made’ [29].

Methodological frameworks are needed to guide diagnostic test evaluation and inform stakeholders about what evidence should be sought to justify implementation. They can be of particular value in areas of uncertainty or high complexity, or where there are differing opinions between stakeholders. We identified specific needs relating to the evaluation of cancer diagnostic tests in low prevalence populations where spectrum bias becomes more important; and where tests are used for triage and incorporated into a diagnostic strategy. While the framework has been developed for cancer detection research, its principles are generic and applicable not only to cancer but also to many other low prevalence conditions.

The framework makes explicit not only *what is being evaluated* at each phase, but crucially, *what is changing* in the PICO elements at each of these phases. Test evaluation may be iterative and the results of one phase may lead test designers back to earlier phases and redesign of the test. Patients are at the heart of this framework, from driving the research agenda and the push for new tests, via careful evaluation of patient-centred outcomes including acceptability, harms and benefits in phase 3, to the evaluation of population outcomes including inequalities, stage shift and survival post-implementation in Phase 5. All these points are strengths.

While the framework encompasses a series of necessary phases, in reality tests may be implemented before evidence exists from Phase 3 and 4 studies, leaving Phase 5 research as the only option. The sample sizes required to show whether detecting low prevalence disease improves patient outcomes (Phase 3 and above) are large and may not be feasible; indeed, modelling studies rather than empirical evaluation may be the only possibility. This may be the case, for example, where a new test is more accurate than an existing test.

Some phases of the framework may require expansion over time: in Phase 3, although we had patient input to the consensus, it is unclear which components or outcomes of test evaluation are most important to patients themselves. This deficit has been recognised by the US-based Patient-Centred Outcomes Research Institute and other groups, and is an area of current research [30]. In Phase 5, we refer to evaluating utilisation of the test across the healthcare system; this implies a focus on implementation research methods and evaluation of effects on workflow, staffing and logistics, which are vital for test sustainability.

A key question with any proposed new framework is ‘How could or should it be used?’ [18]. The CanTest Framework helps map current evidence about a test, and where that sits along the Phases of test evaluation. This supports decisions about the next critical research questions and future study designs. Examples of cancer detection tests in low prevalence populations to which application of the CanTest Framework would be useful have been illustrated in Table 2 and include:

(i) existing tests transferred to primary care, or to point-of-care, for detecting specific cancers among defined groups in primary care populations, including those with and without symptoms (e.g. Faecal Immunochemical Testing for colorectal cancer);
(ii) existing tests currently only accessible in secondary care being made more available to primary care practitioners (e.g. CT or MRI scans);
(iii) novel tests with potential relevance for primary care, such as the Stockholm-3 model for prostate cancer detection [31], or the CancerSEEK biomarker panel [32].

**Table 2:** Real-world examples, applying the CanTest Framework

Phases of the CanTest Framework	POPULATION	TEST	COMPARATORS	OUTCOMES	Examples		
					CA125 for detecting ovarian cancer	CytoSponge™ for detecting Barrett’s Oesophagus (at high risk for oesophageal adenocarcinoma)	CancerSEEK biomarker panel for detecting 8 common cancer types
*DEFINITIONS/background*					Cancer Antigen (CA)125 is a serum biomarker for epithelial ovarian cancer. It is utilized in strategies to distinguish benign from malignant pelvic masses pre-surgery and in the triage of women in primary care. It has been evaluated as part of screening strategies but is not currently used in that setting.	A non-endoscopic ‘sponge on a string’ test, used for the diagnosis of oesophageal squamous carcinomas in high-risk areas, was adapted for Barratt’s Oesophagus (BO) by combining it with immunocytology	A blood test to detect 8 common cancer types through assessment of the levels of circulating proteins and mutations in cell-free DNA
*Phase 1 Selection of test and initial measures of single test performance*	Highly selected	single	Reference standard	Performance			
Analytic validity					Multiple studies e.g. Bast *et al*. 1983^a^: assay developed and threshold set (> 35 U/ml); 1% of healthy patients and 82% of patients with ovarian carcinomas have levels > 35 U/ml. Mongia *et al*. 2006^b^: Comparison of 6 CA125 assays; acceptable performance and comparability.	Lao-Sirieix *et al*. 2009^j^: Trefoil factor 3 (TFF3) expressed to high levels in BO compared to normal oesophagus or gastric mucosa; sensitivity 78%, specificity 94%	Cohen *et al*. 2018^n^: For non-metastatic cancers: sensitivity 69–98% for 5 cancer types; specificity > 99%
Diagnostic accuracy					Multiple studies e.g. Jacobs *et al*. 1989^c^: Pooled sensitivity for ovarian cancer 85%.	N/A	N/A
*Phase 2 Measures of clinical test performance*	Highly selecte	Single	Reference	performance			
Diagnostic accuracy	d				Multiple studies e.g. Maggino *et al*. 1994^d^: Sensitivity 78.3%, specificity 82% for ovarian cancer in patients with a pelvic mass.	Ross-Innes *et al,* 2015^k^: Sensitivity 79.9%, specificity 92.4% for BO in patients referred with dyspepsia and reflux symptoms.	N/A
Internal validity / reproducibility					Multiple studies e.g. Medeiros *et al*. 2009^e^: systematic review, Area Under the Curve of 0.9 for distinguishing malignant/borderline and benign tumours.	N/A	N/A
*Phase 3 Impact on clinical decision-making & health outcomes*	Selected/Real-world	Single/combinations	Reference/ usual care	Medical decision making			
Diagnostic accuracy					N/A	Kadri *et al,* 2010^l^. Accuracy for BO in primary care: sensitivity 90% & specificity 93.5% for clinically relevant segments of 2 cm or more compared with gastroscopy.	N/A
Effects on patients					N/A	Kadri *et al,* 2010^l^. Acceptable for patients, and no adverse events.	N/A
Effects on clinicians					Moss *et al*. 2013^f^: Explored GP views on CA125 use in Primary care.		N/A
Effects on diagnostic triage / Incorporation into diagnostic strategies					Gilbert *et al*. 2012^g^: Pilot study of symptom triggered ‘screening’ strategy incorporating CA125 and ultrasound. Study arm patients had more frequently resectable tumours than the control arm (usual care). Definitive results awaited.	N/A	N/A
*Phase 4 Effectiveness of new diagnostic strategy on clinical outcomes*	Real-world	Single/combinations	Usual care	MDM/harms			
Effectiveness & cost-effectiveness					NICE 2011^h^: cost effectiveness comparison of different triaging strategies incorporating CA125.	Offman *et al,* 2018^m^: BEST3 randomised trial underway comparing the Cytosponge-TFF3 test with usual care to facilitate diagnosis of oesophageal pre-cancer in primary care patients with chronic acid reflux.	N/A
Patient safety & quality					Goff *et al*. 2012^i^: Small study; symptom based testing in primary care resulted in minimal additional unnecessary procedures.	N/A	N/A
Over-diagnosis					N/A	N/A	N/A
*Phase 5 Implementation & effects at healthcare & population level*	Real-world			Pop health & costs			
Effects on health system					N/A	N/A	N/A
Effects on population					N/A	N/A	N/A

^a^ Bast *et al*. 1983: https://www.nejm.org/doi/full/10.1056/NEJM198310133091503?url_ver=Z39.882003&rfr_id=ori%3Arid%3Acrossref.org&rfr

^b^ Mongia *et al*. 2006: https://www.ncbi.nlm.nih.gov/pubmed/16690492

^c^ Jacobs *et al*. 1989: https://academic.oup.com/humrep/article/4/1/1/608701

^d^ Maggino *et al*. 1994: https://www.sciencedirect.com/science/article/pii/S0090825884711796

^e^ Medeiros *et al*. 2009: https://www.ncbi.nlm.nih.gov/pubmed/18995946

^f^ Moss *et al*. 2013: https://www.ncbi.nlm.nih.gov/pmc/articles/PMC3644283/

^g^ Gilbert *et al*. 2012: https://www.thelancet.com/journals/lanonc/article/PIIS1470-2045(11)70333-3/fulltext

^h^ NICE. Ovarian Cancer: The recognition and initial management of ovarian cancer. Cardiff, UK: National Collaborating Centre for Cancer, 2011

^I^ Goff *et al*. 2012: https://www.sciencedirect.com/science/article/pii/S0090825811008742

^J^ Lao-Sirieix *et al*. 2009: doi: 10.1136/gut.2009.180281

^K^ Ross-Innes *et al*. 2015: doi: 10.1371/journal.pmed.1001780

^L^ Kadri *et al*. 2010: doi: 10.1136/bmj.c4372

^M^ Offman *et al*. 2018: doi: 10.1186/s12885-018-4664-3

^N^ Cohen et al.2018: https://www.ncbi.nlm.nih.gov/pubmed/29348365

The CanTest Framework acknowledges the need to address cognitive and cultural factors influencing decision-making about diagnostic testing during the medical consultation, coordination of the diagnostic process (often involving performance and interpretation of different tests at different times and locations), and fail-safe patient follow-up [3, 23, 33]. These are all critical to research in Phases 3–5 to understand how tests are incorporated into diagnostic strategies and how they are implemented into routine practice.

This new framework has relevance for a wide range of stakeholders. Diagnostic test developers have traditionally focused on the earlier phases of research examining analytical accuracy and preliminary evidence of performance characteristics [15], while health services researchers have focused on studies of effectiveness and implementation in various clinical settings. Researchers from these different perspectives will find that the framework guides their research and design strategies and choice of outcomes. It may also promote collaboration across researcher ‘silos’ to promote speedier translation of promising tests from bench to clinic.

Diagnostic test developers are at the vanguard of technological advances, producing a growing array of new medical diagnostics [34]. However, they often find that return on investment for new tests is low, and there may be little commercial incentive to support clinical testing beyond requirements for regulatory approval. We anticipate that the CanTest Framework can promote collaboration and partnership between industry, academia and healthcare providers to undertake phased evaluation, aiming to deliver a product whose benefits outweigh its harms. Furthermore, it will be useful to facilitate a shared language for communication between test developers and clinicians about what evidence is needed before considering promoting it for routine clinical use. It may be particularly useful to better inform or combat speculative media reports of early stage biomarker research like ‘a promising new test to detect cancer early’ [32], when the framework will demonstrate that a test may only be in an early phase of evidence gathering.

Clinicians need trustworthy evidence about the value of a test, as well as information as to how it fits best into diagnostic strategies and approaches in daily practice, its role in decision-making and triage, and its effects on patient safety [16]. Patients may assume that all cancer testing is inherently beneficial, without being aware of the potential harms from false positive results, over-detection of slow-growing cancers and subsequent overtreatment. Generating evidence that patients can use with their clinicians, to weigh up the potential harms and benefits of a tests is highly valuable [30].

Policy makers also may feel pressure from various groups to approve, or reimburse, new tests, particularly in areas such as cancer that are often in the public eye. The framework provides a way that policy makers and health technology assessment groups can use to assess how far along the development to implementation pathway a new test is, and guide deployment decisions. Finally, funders have traditionally struggled to encourage collaborations between academic and commercial groups. Adopting the CanTest Framework would enable funding bodies (and also test developers) to specify exactly where and how they wish to drive the early detection and diagnosis research agenda.

## Conclusion

A robust conceptual approach to development, implementation and evaluation of cancer diagnostic tests would ensure that they are fit for purpose when introduced into clinical practice. Because no suitable framework is currently available, we developed the CanTest Framework to address this gap, proposing that this will help to overcome methodological and practical challenges to improve decision making and patient outcomes related to diagnostic tests [35]. The new framework presents an advance that addresses specifically the evaluation of cancer diagnostic tests along the continuum from test development to routine use in the intended population. Although focused specifically on cancer diagnostics, it is applicable to the development and evaluation of many diagnostic and screening tests which are intended for use in low prevalence populations.

## Additional file

Additional file 1: **Table S1.** Included Frameworks. (DOCX 35 kb)

## Data Availability

All data generated or analysed during this study are included in this published article and its supplementary information files.

## Abbreviations

BMI: Body Mass Index
CA125: Cancer Antigen 125
CT: Computed tomography
IQR: InterQuartile Range
MRI: Magnetic resonance imaging
PSA: Prostate Specific Antigen
UK: United Kingdom
US: United States
