# Supplementary material for: Evaluating diagnostic strategies for early detection of cancer: the CanTest framework
Source: BMC Cancer. 2019 Jun 14;19:586. doi: 10.1186/s12885-019-5746-6 (PMC6570853; doi:10.1186/s12885-019-5746-6)
Supplement: Supplementary file 1 — Table S1. Included Frameworks. (DOCX 35 kb) [file 12885_2019_5746_MOESM1_ESM.docx]

**Additional file 1: Table S1 Summary of Included Frameworks**

(N/S = not specified but implied)

|  | **Framework**  **Authors, year, setting** | **Field** | **Cancer-specific** | **Low prevalence population** | **Triage test** | **Diagnostic strategy** | **Summary of framework** |
| --- | --- | --- | --- | --- | --- | --- | --- |
| **GENERIC TEST FRAMEWORKS n=8** | | | | | | | |
| **1** | ***Hierarchical Model of Efficacy***  **Fryback & Thornbury**, ^12^ 1991, USA | Imaging | No | No | Yes | No | Describes six hierarchical tiers of diagnostic efficacy: technical, diagnostic, diagnostic thinking, therapeutic, patient outcome, and societal.  (Indirectly considers costs and the cost-effectiveness of a technology).  Advocates RCTs for tests that have a higher risk of harm, are more expensive, and/or have wide utilization. Highly cited paper in subsequent frameworks. |
| **2** | ***U.S. Preventive Services Task Force***  **Harris et al.** ^14^  2001, USA | Generic | No | Yes | No | N/S | Identifies the populations, preventive services, diagnostic or therapeutic interventions, and intermediate and health outcomes being considered for a screening test. Requires a “chain of logic” in which evidence must support to link the preventive service to improved health outcomes. Content is considered alongside the quality of supporting evidence and weighing of benefits and harms. |
| **3** | ***Phases of biomarker development for early detection of cancer.***  **Pepe et al.**  2001, USA | Laboratory medicine | Yes | No | No | No | Proposes guidelines to facilitate the development of biomarker-  based screening tools for early detection of cancer for the NCI’s Early Detection Research Network (EDRN). Categorizes development into  five phases that a biomarker needs to pass through ‘to produce a  useful population-screening tool’. |
| **4** | ***Framework for assessing the vaue of diagnostic imaging***  **Gazelle et al.** ^15^  2011**,** USA | Imaging | No | No | No | N/S | Aims to ‘provide guidance for researchers, manufacturers, regulators, and reimbursement decision makers about relevant outcomes data for assessing the value of diagnostic imaging technologies.’  Builds on Fryback & Thornbury’s framework, adding three characteristics: size of the at-risk population, anticipated clinical impact, and potential economic impact. |
| **5** | ***Evaluating the clinical utility of tumor markers in oncology.***  **NCCN Task Force Report**  **Febbo et al.** ^13^  2011, USA | Oncology | Yes | No | No | No | Aims to ‘codify the appropriate evaluation of the required level of evidence necessary for making recommendations for incorporating new tests into clinical practice guidelines and general clinical use.’  Provides definitions and regulatory context in the evaluation of analytic validity, clinical validity, and clinical utility of a tumor marker. Proposes the use of a combination level of evidence score to in the evaluation of biomarker tests. |
| **6** | ***Framework for designing and evaluating trials.***  **Ferrante Di Ruffano et al.** ^16^  2012, UK and international partners | Generic | No | No | Yes | Yes | Aims to ‘Present a framework for the design and interpretation of studies that evaluate the health consequences of new diagnostic tests.’  Includes linear test – treatment pathway which seeks a systemic approach to evaluating tests as a complex intervention. Emphasis on patient outcomes and influence on decisions that influence diagnostic and treatment decisions, affecting time to treatment, modifying patient perceptions and behaviour, or putting patients at risk of harm. Includes a summary checklist to assist in design or appraisal of studies evaluating diagnostic tests. |
| **7** | ***From biomarkers to medical tests: The changing landscape of test evaluation.***  **Horvath et al.** ^17^  2014, Australia | Laboratory medicine | No | No | Yes | Yes | For the Test Evaluation Working Group of the European Federation of Clinical Chemistry Laboratory Medicine, aims to provide ‘guidance and practical tools for assessing the clinical beneﬁts of in vitro medical tests.’  A cyclical framework with key components of the test evaluation process: (1) Analytical performance, (2) Clinical performance, (3) Clinical effectiveness, (4) Comparison of effectiveness between tests, (5) cost-effectiveness, (6) Broader impact (e.g., acceptability, social, psychological, legal, ethical, societal and organisational consequences). |
| **8** | ***Framework to incorporate multiple test attributes in evaluating diagnostic tests including Point-of-Care tests.***  **Thompson et al.** ^18^  2016, USA | Generic | No | Yes | N/S | N/S | Aims to provide ‘an approach which potentially allows multiple stakeholder preferences to be incorporated into the assessment of benefits and risks of diagnostic tests.’  Designed for clinical settings, it shows interactions between test attributes (test availability, test experience, and test results) and outcomes. Considers balance / trade-offs between potential benefits and harms. Focuses on patients and the often non-linear interactive nature of components of the model. |
| **TESTS SPECIFIC TO EVAULATING GENETIC TESTS n=6** | | | | | | | |
| **9** | ***Diagnostics and biomarker development: priming the pipeline.***  **Phillips et al.** ^4^  2006 USA | Pharmacogenetics | No | No | No | No | Aims to ‘address the pipeline problem for diagnostics and biomarkers, and the unique aspects distinguishing the problems in diagnostic and drug development.’  Describes phased approach to co-developing and regulating combined test-drug products. Includes following phases: marker assay validation, analytical validation, pre-clinical feasibility, clinical validation and clinical utility. |
| **10** | ***Evaluation of Genomic Applications in Practice and Prevention- the EGAPP Framework.***  **Teutsch et al.** ^19^  2009, USA | Genetics | No | Yes | No | No | Aims to ‘develop a systematic process for evidence-based assessment, specifically focused on genetic tests and other applications of genomic technology.’  Superseded the ACCE Framework from 2004 onwards, and based on the ACCE criteria, the US Preventive Services Task Force, and some Fryback-Thornbury model components. Focuses on clinical factors and health-related outcomes, key questions, and methodological models, and information on a test’s impact on issues such as management decisions by physicians and patients, and on the cost-effectiveness and feasibility of the use of the test. |
| **11** | ***The Contribution of Health Technology***  ***Assessment, Health Needs Assessment, and***  ***Health Impact Assessment to the Assessment and***  ***Translation of Technologies in the Field of Public***  ***Health Genomics****.*  **Rosenkötter et al.** ^20^  2011, European | Genetics | No | N/S | No | No | Aims to assess ‘approaches in public health, like health technology assessment (HTA), health needs assessment (HNA) and health impact assessment (HIA) and their contribution to the translation of genome-based health applications.’  Comprises a critical analysis of the evidence on the analytical and clinical validity of the technology, an assessment of the utility, acceptability and feasibility of the diagnostic and screening strategies as well as an organisational analysis of the technology’s interaction with health care delivery and services. Ethical, legal and social issues emerge at all stages of the analysis. |
| **12** | ***Development and description of GETT: a Genetic testing Evidence Tracking Tool.***  **Rousseau et al.** ^21^  2010**,** Canada | Genetics | No | N/S | N/S | No | An operational tool, intended to build on existing frameworks. Consists of a checklist of 72 questions and their definitions that should be considered when evaluating genetic tests. Checklist categorized into 10 major themes: overview of the disease, diagnostic tool, quality improvement programs, clinical utility, diagnostic and screening strategies, impacts on health care system, psychological and societal impacts, ethical and legal impacts, synthesis of information — including missing data, and identification of research priorities.  The tool incorporates areas typically absent from most models, such as the availability of quality improvement/proficiency programs and the availability and accessibility of professional services, health care and follow-up, and expertise and training. |
| **13** | ***Evaluation frameworks and assessment of analytic validity.***  **Sun et al.** ^22^  2013, USA | Genetics | No | N/S | No | N/S | Proposes a set of evaluations frameworks for seven testing scenarios based on an adaptation of the EGAPP framework: diagnosis in symptomatic patients; disease screening in asymptomatic patients; prognosis assessment; treatment monitoring; drug selection (including pharmacogenetics); risk/susceptibility assessment, and testing for germline-mutation-related conditions. |
| **14** | ***Evaluating genomic tests from bench to bedside: a practical framework.***  **Lin et al.** ^23^  2012, USA, UK | Genetics | No | Yes | No | N/S | Aims to describe a ‘framework for assessing the evidence base for genomic tests (from discovery to clinical adoption) that builds upon existing published frameworks for evaluating genomic tests, without necessarily specifying any particular threshold of evidence for regulatory approval or clinical implementation.’  Organized around six phases in the development of genomic tests beginning with marker identification and ending with population impact, and highlights knowledge gaps in establishing the clinical relevance of a test. Framework focuses on the clinical appropriateness of the four main dimensions of test research questions (population/setting, intervention/index test, comparators / reference test, and outcomes). |
| **OTHER FRAMEWORKS n=2** | | | | | | | |
| **15** | ***Safer Dx Framework.***  **Singh** **& Sittig,** ^24^  2015, USA | Generic | No | N/S | Yes | Yes | Aims to ‘provide a conceptual foundation for system-wide safety measurement, monitoring and improvement.’  Draws on Donabedian’s Quality of Care (Structure, Process, Outcome), and can be used for measurement, monitoring and improvement of diagnostic error. The framework “accounts for the complex adaptive sociotechnical system in which diagnosis takes place (the structure), the distributed process dimensions in which diagnoses evolve beyond the doctor’s visit (the process) and the outcomes of a correct and timely “safe diagnosis” as well as patient and health care outcomes (the outcomes).” |
| **16** | ***Anatomy of value proposition for laboratory medicine.***  **Price & St John,** ^25^  2014, UK & international partners | Laboratory medicine | No | N/S | Yes | Yes | Provides a checklist for developing a value proposition in laboratory medicine listing the issues that need to be addressed at each stage of the value proposition and the key questions that need to be answered in a logical sequence. |
